# Supplementary material for: Sleepiness and Fatigue as Consequences of Cumulative Sleep Restriction: Insights from Fine-Grained Subjective Measures and Skin Temperature in the Field
Source: Clocks Sleep. 2025 Sep 19;7(3):51. doi: 10.3390/clockssleep7030051 (PMC12452710; doi:10.3390/clockssleep7030051)
Supplement: Supplementary file 1 [file clockssleep-07-00051-s001.zip › clockssleep-3737793-supplementary.pdf]

Supplementary Material

Table S1.
Descriptive statistics of the sleep metrics within respective conditions (baseline, normal sleep, and restricted sleep; data extracted from sleep diaries).

Condition	Sleep metrics	Mean	std	min	max
Baseline	Sleep duration (hours)	7.99	1.16	3.47	12.25
	Time in bed (hours)	8.73	1.30	5.62	14.00
	Sleep latency (min)	14.88	15.88	0	120
	Morning stay in bed (min)	9.74	16.64	0	120
	Sleep quality (0-4)	2.65	0.81	1	4
	Restfulness (0-5)	2.37	0.80	0	4
	Sleep onset (time)	00:17	01:25	20:08	04:00
	Sleep offset (time)	08:15	01:35	02:25	11:45
Normal sleep	Sleep duration (hours)	8.18	0.77	6.67	9.67
	Time in bed (hours)	8.90	1.06	7.20	11.50
	Sleep latency (min)	13.10	8.26	1	45
	Morning stay in bed (min)	10.33	16.61	0	90
	Sleep quality (0-4)	2.80	0.61	1	4
	Restfulness (0-5)	2.57	0.61	1	4
	Sleep onset (time)	23:54	00:59	21:40	02:30
	Sleep offset (time)	08:05	01:07	06:00	10:48
Restricted sleep	Sleep duration (hours)	4.06	0.34	3.17	5.75
	Time in bed (hours)	4.78	0.95	4.00	8.33
	Sleep latency (min)	8.80	6.94	1	30
	Morning stay in bed (min)	5.84	11.68	0	60
	Sleep quality (0-4)	2.52	0.84	1	4
	Restfulness (0-5)	1.12	0.80	0	3
	Sleep onset (time)	03:16	00:43	02:00	04:55
	Sleep offset (time)	07:19	00:43	06:00	09:00

Table S2.

Estimated marginal means and standard error of the momentary sleepiness (KSS, SSS) and momentary fatigue ($VAS_{fatigue}$) per day and per condition.

	KSS		SSS		VAS	
	NS	RS	NS	RS	NS	RS
Day 1	3.19 ± 0.21	4.06 ± 0.27	2.51 ± 0.15	3.10 ± 0.17	3.14 ± 0.35	4.29 ± 0.35
Day 2	3.10 ± 0.21	4.45 ± 0.27	2.47 ± 0.16	3.32 ± 0.17	2.92 ± 0.35	4.73 ± 0.35
Day 3	3.08 ± 0.21	4.83 ± 0.28	2.70 ± 0.16	3.81 ± 0.17	3.11 ± 0.36	5.23 ± 0.36

Note. NS: Normal sleep; RS: Restricted sleep.

Table S3.

Summary of statistics of post-hoc contrast analyses on the interaction term Condition * Day's effect on Karolinska sleepiness scale score (KSS).

**Contrast between Condition within Day**		**Estimate**	**Standard error**	**P value**
Day 1	NS-RS	-0.87	0.27	<.01
Day 2	NS-RS	-1.35	0.27	<.001
Day 3	NS-RS	-1.76	0.28	<.001
**Contrast between Day within Condition**				
Normal sleep	Day1 - Day2	0.09	0.17	0.87
	Day1 - Day3	0.11	0.17	0.79
	Day2 - Day3	0.03	0.17	0.99
Restricted sleep	Day1 - Day2	-0.39	0.16	0.03
	Day1 - Day3	-0.77	0.17	<.001
	Day2 - Day3	-0.38	0.17	0.06

Note. NS= Normal sleep; RS= Restricted sleep.

Table S4.

Summary of statistics of post-hoc contrast analyses on the interaction term Condition * Day's effect on Stanford sleepiness scale score (SSS).

**Contrast between Condition within Day**		**Estimate**	**Standard error**	**P value**
Day 1	NS-RS	-0.60	0.19	<.01
Day 2	NS-RS	-0.85	0.19	<.001
Day 3	NS-RS	-1.11	0.20	<.001
**Contrast between Day within Condition**				
Normal sleep	Day1 - Day2	0.04	0.13	0.96
	Day1 - Day3	-0.19	0.13	0.32
	Day2 - Day3	-0.23	0.13	0.20
Restricted sleep	Day1 - Day2	-0.22	0.12	0.15
	Day1 - Day3	-0.70	0.13	<.001
	Day2 - Day3	-0.49	0.13	<.001

Note. NS= Normal sleep; RS= Restricted sleep.

Table S5.

Summary of statistics of post-hoc contrast analyses on the interaction term Condition * Day's effect on Visual analogue scale score ($VAS_{fatigue}$).

Contrast between Condition within Day		Estimate	Standard error	P value
Day 1	NS-RS	-1.15	0.31	<.001
Day 2	NS-RS	-1.81	0.31	<.001
Day 3	NS-RS	-2.12	0.32	<.001
**Contrast between Day within Condition**				
Normal sleep	Day1 - Day2	0.22	0.20	0.51
	Day1 - Day3	0.03	0.21	0.98
	Day2 - Day3	-0.19	0.21	0.63
Restricted sleep	Day1 - Day2	-0.44	0.19	0.05
	Day1 - Day3	-0.94	0.20	<.001
	Day2 - Day3	-0.50	0.20	0.03

Note. NS= Normal sleep; RS= Restricted sleep.

Table S6.

Summary of statistics on the relation between features of momentary sleepiness (KSS or SSS) or momentary fatigue ($VAS_{fatigue}$) (all three person mean centered) and daily fatigue (PROMIS). Models included Participant as a random intercept.

		F	p	B	SE	ß	CI
KSS	Mean*	$F_{1/12}$= 19.96	**<.001**	3.91	0.87	0.69	[0.35, 1.03]
	Min.	$F_{1/80}$= 20.06	**<.001**	2.62	0.59	0.46	[0.26, 0.67]
	Max.	$F_{1/75}$= 26.38	**<.001**	1.67	0.33	0.49	[0.30, 0.68]
	First	$F_{1/84}$= 3.89	0.05	0.67	0.34	0.22	[0.00, 0.45]
	Last	$F_{1/81}$= 24.17	**<.001**	1.58	0.32	0.52	[0.31, 0.72]
SSS	Mean*	$F_{1/13}$= 24.33	**<.001**	5.37	1.09	0.66	[0.37, 0.94]
	Min.	$F_{1/81}$= 22.73	**<.001**	3.91	0.82	0.50	[0.29, 0.70]
	Max.	$F_{1/78}$= 29.93	**<.001**	2.30	0.42	0.53	[0.33, 0.72]
	First	$F_{1/83}$= 11.24	**<.01**	1.61	0.48	0.37	[0.15, 0.59]
	Last	$F_{1/82}$= 12.40	**<.001**	1.46	0.41	0.38	[0.17, 0.60]
$VAS_{fatigue}$	Mean	$F_{1/71}$ = 49.43	**<.001**	2.65	0.38	0.59	[0.42, 0.76]
	Min.	$F_{1/85}$ = 8.16	**<.01**	1.47	0.51	0.33	[0.10, 0.56]
	Max.	$F_{1/77}$ = 32.06	**<.001**	1.52	0.27	0.53	[0.35, 0.72]
	First	$F_{1/81}$ = 14.81	**<.001**	1.16	0.30	0.40	[0.19, 0.61]
	Last	$F_{1/84}$ = 9.29	**<.01**	0.86	0.28	0.33	[0.12, 0.55]

Note. F= F statistics, p= p-value, KSS= Karolinska Sleepiness Scale, SSS= Stanford sleepiness scale, VAS= visual analogue scale of fatigue, B: parameter estimate, SE= standard error, ß= standardized parameter, CI= confidence interval. Statistically significant associations (p <.01) are presented in bold. * Random slope was added as it improved model fit.

Table S7.

Demographic information and sleep durations

	Age	PSQI	MSF
**Mean**	22.24	3.65	4.90
**SD**	3.73	1	0.79
**Min**	19	2	3.38
**Max**	32	5	6.13

Note. PSQI= Pittsburg sleep quality index; MSF= Mid sleep on free day, time in decimals.

Table S8.

Intraclass correlation (ICC) in unconditional models.

	**ICC** (Participant)	**ICC** (Session within Participant)
**KSS**	0.20	
**SSS**	0.14	
**VAS $_{fatigue}$**	0.11	0.34
**ESS**	0.18	
**PROMIS**	0.14	
**DST**	0.26	
**PST**	0.28	0.10
**DPG**	0.29	0.04

Note. KSS= Karolinska sleepiness scale; SSS= Stanford sleepiness scale; $VAS_{fatigue}$= Visual analogue scale of fatigue; ESS= Epworth sleepiness scale; PROMIS= Patient-Reported Outcomes Measurement Information System on fatigue; DST= Distal skin temperature; PST= Proximal skin temperature; DPG= Distal to proximal gradient.
